# Different epigenetic signatures of newborn telomere length and telomere attrition rate in early life

**DOI:** 10.18632/aging.203117

**Published:** 2021-06-04

**Authors:** Congrong Wang, Tim S. Nawrot, Charlotte Van Der Stukken, Dominika Tylus, Hanne Sleurs, Martien Peusens, Rossella Alfano, Sabine A.S. Langie, Michelle Plusquin, Dries S. Martens

**Affiliations:** 1 Centre for Environmental Sciences, Hasselt University, Hasselt, Belgium; 2 Department of Public Health and Primary Care, Leuven University, Leuven, Belgium; 3 Department of Pharmacology and Toxicology, School for Nutrition and Translational Research in Metabolism (NUTRIM), Maastricht University, Maastricht, The Netherlands

**Keywords:** telomere length, telomere attrition, DNA methylation, early life, newborn

## Abstract

Telomere length (TL) and telomere shortening are biological indicators of aging, and epigenetic associates have been found for TL in adults. However, the role of epigenetic signatures in setting newborn TL and early life telomere dynamics is unknown. In the present study, based on 247 participating newborns from the ENVIR*ON*AGE birth cohort, whole-genome DNA methylation, profiled on the Illumina MethylationEPIC BeadChip microarray, and TL were measured in cord blood. In a follow-up visit at a mean age of 4.58 years, leukocyte TL was evaluated. We combined an epigenome-wide association study and a statistical learning method with re-sampling to select CpGs and their two-way interactions to model baseline (cord blood) TL and early-life telomere attrition rate, where distinct epigenetic signatures were identified for the two outcomes. In addition, a stronger epigenetic regulation was suggested in setting newborn TL than that of telomere dynamics in early life: 47 CpGs and 7 between-CpG interactions explained 76% of the variance in baseline TLs, while 72% of the total variance in telomere attrition rate was explained by 31 CpGs and 5 interactions. Functional enrichment analysis based on the selected CpGs in the two models revealed GLUT4 translocation and immune cell signaling pathways, respectively. These CpGs and interactions, as well as the cellular pathways, are potential novel targets of further investigation of telomere biology and aging.

## INTRODUCTION

Telomeres are protective nucleoprotein caps built up from tandem repeats of the hexamer sequence “TTAGGG” at the ends of chromosomes which is crucial for chromosomal stability [1]. Telomeres progressively shorten after each cellular division, owing to the incomplete replication of DNA molecules at the end of chromosomes (referred as the end-replication problem) and telomere maintenance mechanisms that are not capable of preventing telomere attrition. Therefore, telomere length reflects the cycles of mitosis a cell has been through and the extent of biological aging. Telomere length in a somatic cells is the longest at birth and telomere attrition occurs throughout the lifespan [2]. Shorter telomere length is an indicator of a higher risk of age-related diseases and a predictor of mortality [3–7]. In addition to cell division, telomere dynamics has been shown to associate with genetic alterations [8], biochemical factors such as oxidative stress [9], socioeconomic status [10], environmental exposures [11–13] and lifestyle factors such as obesity [14].

Another widely studied molecular marker of the biology of aging is DNA methylation. DNA methylation in human cells is a modification of DNA molecules by the addition of methyl groups, in the most widely studied context, to cytosine residue in cytosine-phosphate-guanines (CpGs) forming 5-methylcytosines, which plays a pivotal role in the regulation of human gene expression [15]. Differences in DNA methylation, similar to telomere length, exhibit strong correlations with aging, longevity and age-related diseases [16, 17]. Studies have identified various sets of CpGs as predictors of human biological age, which formed the epigenetic age clocks [18–20], for wide-ranged age groups as well as specifically for newborns [21, 22]. The difference between epigenetic age and chronological age measures biological aging status [23].

There have been investigations in the link between these two biomarkers of aging. A population-based study [24] elucidated that DNA methylation at specific sub-telomeric CpGs and imprinted loci were associated with telomere length. A DNA methylation-based predictor of telomere length in adults has been developed and validated in different tissues and cell types which predicted mortality and aging-related diseases [25]. However, despite that telomere length in early life is an important determinant of telomere length in adulthood [26, 27] and shows high variation across newborns [28], currently no study has examined the association between telomere length and DNA methylation in newborns. The regulation of telomere length at early stage of life might involve DNA methylation at different loci compared to those in adulthood, as both telomere length and DNA methylation are dynamic processes over life [2, 29]. Furthermore, no study yet has reported such findings in a longitudinal setting allowing for telomere tracking from birth to young childhood.

We hypothesized that newborn genome-wide DNA methylation is associated with telomere length at birth and telomere attrition rate in early life. The present study was performed on a subset of mother-newborn pairs from the ENVIRonmental influence *ON* AGEing in early life (ENVIR*ON*AGE) birth cohort [30]. We aimed to identify CpGs and CpG-CpG interactions from whole-genome DNA methylation profiles that jointly explained the variation in cord blood telomere lengths and telomere attrition from birth to childhood (age of 4 years), respectively. The identified CpGs were mapped to biological pathways to facilitate our understanding in the potential underlying epigenetic mechanisms of early-life telomere biology.

## RESULTS

### Study population

Table 1 shows the demographic characteristics of the mother-newborn (mother-child) pairs. The 247 newborns/children had a mean (standard deviation, SD) gestational age of 39.24 (1.37) weeks, and age of 4.58 (0.39) years at follow-up. The study population included 121 (49.0%) girls, and 93.5% of newborns were of European-origin. The mean (SD) cord blood telomere length, as a surrogate for baseline telomere length, was 1.17 (0.27) T/S ratio. The leukocyte telomere length of the children was 1.02 (0.19) T/S and on average 0.14 T/S shorter than cord blood telomere length (p<0.0001). The variations in cord blood telomere lengths and leukocyte telomere lengths were significantly different (p<0.0001). Telomere attrition corrected for regression-to-the-mean (RTM) effect was on average 0.0025 (0.14) T/S ratio, and telomere attrition rate was on average 0.00072 (0.030) T/S per year.

**Table 1:** Characteristics of the study population (n=247).

		**N (%) or Mean ± SD ^i^**
***Newborns***		
*Sex*	Female	121 (49.0%)
*Ethnicity*	European	231 (93.55)
*Birth weight (kg)*		3.39 ± 0.44
*Gestational age (weeks)*		39.24 ± 1.37
*Age at follow-up (years)*		4.58 ± 0.17
*Telomere length (T/S ratio)*		
	Cord blood	1.17 ± 0.27
	Leukocyte	1.02 ± 0.19
	Telomere attrition ^ii^	0.0025 ± 0.14
Telomere attrition rate (T/S per year) ^iii^		0.00072 ± 0.030
***Mothers***		
*Educational level ^iv^*		
	No diploma	24 (9.75%)
	High school diploma	67 (27.1%)
	A 3-year college	124 (50.2%)
	A 4-year college or university	32 (13.0%)
*Smoking status*		
	Never smoker	167(67.6%)
	Former smoker	48 (19.4%)
	Smoker	32 (13.0%)
*Parity*		
	Primiparous	129 (52.2%)
	Secundiparous	95 (38.5%)
	Multiparous	23 (9.3%)
*With pregnancy complications*		37 (15.0%)
*Age at delivery (years)*		30.21 ± 4.38
*Pre-pregnancy BMI (kg/m^2^)*		24.28 ± 4.56
***Fathers***		
*Age at child birth (years)*		32.87 ± 5.60

^i^Categorical variables were summarized with frequency (N) and percentage (%), while continuous variables were summarized as mean and standard deviation (SD).

^ii^Telomere attrition was calculated as the raw difference of cord blood telomere length and leukocyte telomere length corrected for regression-to-the-mean effect.

^iii^Telomere attrition rate was calculated as telomere attrition divided by the children’s age at follow-up visit.

^iv^Educational level was defined as the highest diploma obtained by the mother.

### The DNAm-based explanatory model of baseline telomere length

### Selection of CpGs

**Figure 1:**
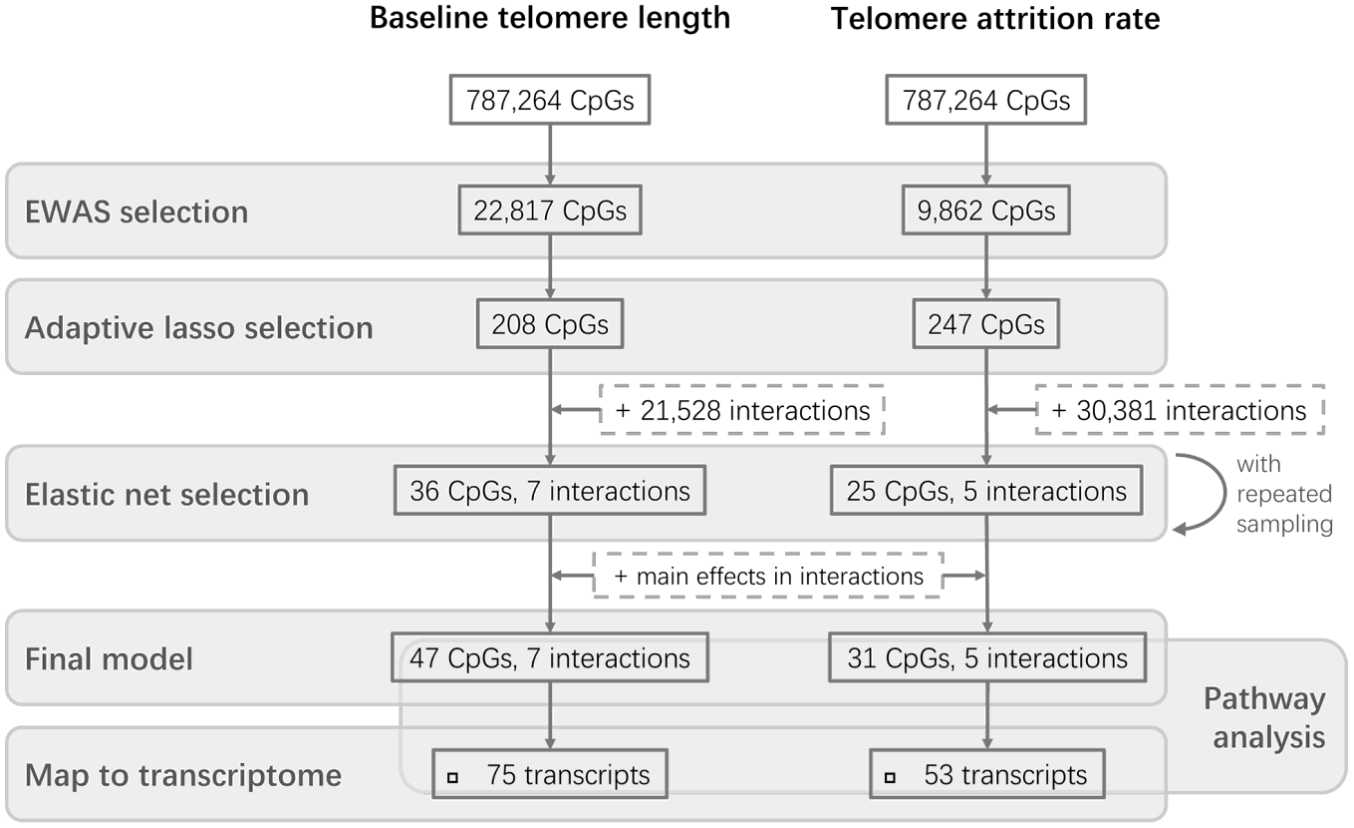
**Flowchart of the study design.** Two parallel stepwise model selections were performed for baseline telomere length and telomere attrition rate, respectively, followed by further investigations of the CpGs in the final models.

**Figure 2:**
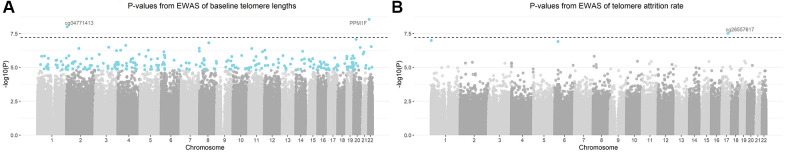
**Manhattan plots of -log10 transformed p-values from the epigenome-wide association analysis (EWAS).** Models were adjusted for gestational age, newborns’ sex, ethnicity and birth weight, maternal age, pre-pregnancy body-mass-index, parity, presence of pregnancy complications, education level and smoking status, paternal age and estimated blood cell counts. CpGs highlighted in blue were significant based on a false discovery rate of 5%. CpG annotated with gene names (or CpG probe names in case that no gene name annotation was available) were the CpGs passing Bonferroni threshold (the dashed horizontal line) of 6.35 × 10^−8^. (**A**) Manhattan plot of baseline telomere length; (**B**) Manhattan plot of telomere attrition rate.

The study design is schematically shown in Figure 1. The epigenome-wide association study (EWAS) of cord blood telomere length, as a pre-screening for candidate CpG predictors from 787,264 CpG probes, identified 244 individual CpGs to be significant (FDR=5%) (Figure 2A). Two CpGs passed the p-value threshold under Bonferroni correction. A loose criterion of p-value<0.01 was applied to allow a sufficiently large number of CpGs in further selection, also considering that CpGs that jointly associate with baseline telomere length might not be the individually significant ones. Therefore, 22,817 CpGs passed the pre-screening and were entered in a subsequent selection in one and same adaptive lasso [31] model which accounts for the between-CpG correlations (Supplementary Table 1). 208 CpGs were jointly selected by adaptive lasso. In order to account for the CpG-CpG interactions, all possible pairwise interactions (21,528 interactions) were added, making it in total 21,736 terms subject to an elastic net selection [32]. In order not to overfit the data, we repeatedly sampled 1000 random subsets from the whole dataset, each containing 85% of the observations (n=210). Variation was observed among the 1000 subsets in terms of the elastic net regularization parameters and the number of selected predictor terms, as shown in Supplementary Figure 1A. Finally, 36 CpG main effects and 7 interactions involving 47 CpGs in total, which were selected in at least 95% of the subsets, were kept as the most relevant. We added the CpGs which were involved in the selected interactions but not in the selected main effects, and thus the final model contained 47 CpG main effects and 7 interactions (54 terms in total).

### The final model and interpretations

**Figure 3:**
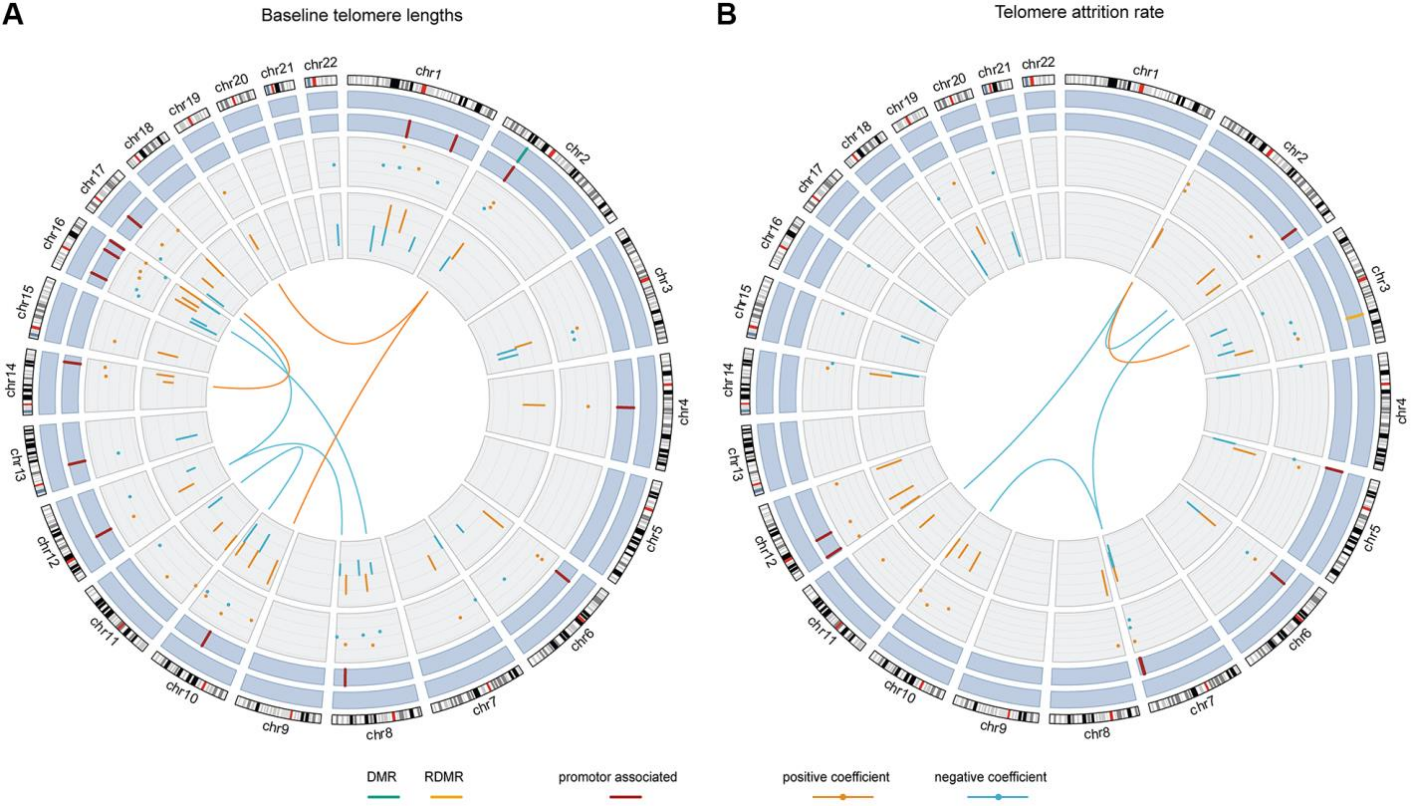
**The chord diagrams depicting the contributing weights (final model coefficients) and EWAS associations of the selected CpGs and CpG-CpG interactions with annotations.** Chromosomes 1 to 22 are displayed clockwise beginning from the top. Tracks from the outside to the inside represent: chromosome ideograms, whether a CpG is involved in known differentially methylated region (DMR, in green) or reprogramming-specific DMR (RDMR, in yellow), whether a CpG is associated with a promotor region (in dark red), the contributing weights in the final models (dots), the epigenome-wide association analysis (EWAS) associations (segments) and the interaction between CpGs, respectively. Panel (**A**) shows the results for baseline telomere length and panel (**B**) for telomere attrition rate. The EWAS coefficients (dots) were shown with dashed grids: in (**A**) the five grids from inside to outside represent -36, -18, 0, 18, 36; in (**B**) the five grids from inside to outside indicate -3, -2, -1, 0, 1, 2. Similarly, the model coefficients (segments) were also shown on dashed grids, with the inner to outside grids indicating value -0.02, -0.01, 0, 0.01, 0.02 in (**A**) and -0.003, -0.002, -0.001, 0, 0.001 and 0.002 in (**B**), respectively. Coefficients larger than 0 are shown in orange and those smaller than 0 are shown in blue.

The Pearson correlation between baseline telomere length and the weighted sum of the 54 terms was 0.89 and the R^2^ was 0.76. Figure 3A and Supplementary Table 2 show the contributing weights (final model coefficients) and the EWAS coefficients of the CpGs and their interactions, as well as the genomic locations. 8 out of the 47 CpG main effects in the final model had been identified significant in EWAS. In the final model, 26 main effect CpGs had a positive coefficient (being positively associated with baseline telomere length) and 21 had a negative coefficient (being inversely associated with baseline telomere length), consistent with their EWAS coefficients. 15 CpGs were promoter-associated and 1 was related to differentially methylated regions (DMRs). The genomic locations of the involved 47 CpGs were distributed across autosomes except chromosomes 5, 9, 18, 20 and 21. All the 7 interaction terms involved CpGs from different chromosomes, among which 3 had positive coefficients indicating that the interactors are mutually promoting their associations with baseline telomere length. The other 4 pairs of interactions with negative coefficients indicated mutual suppression of the associations between their methylation level and baseline telomere length.

**Figure 4:**
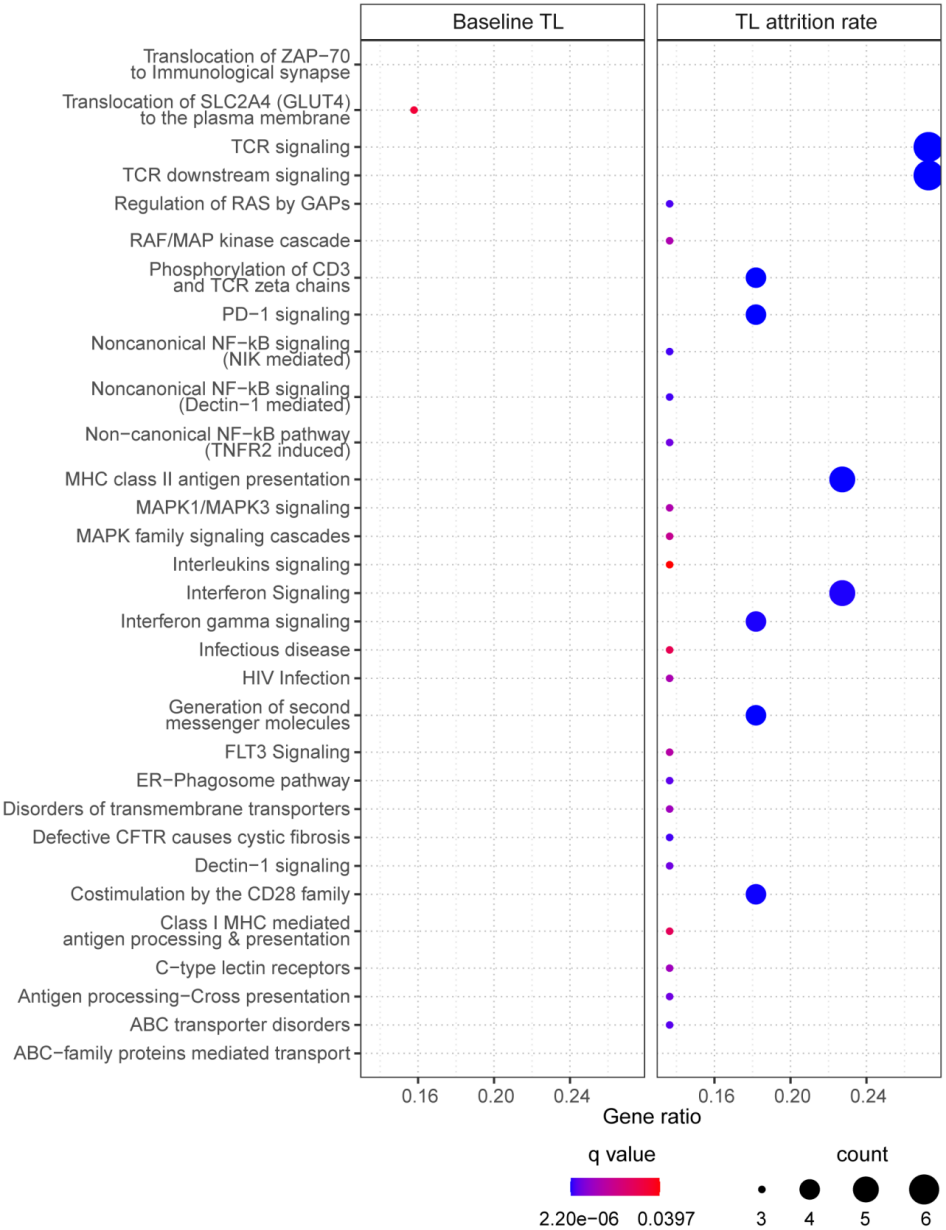
**The enriched pathways from the selected CpGs.** For baseline telomere length (TL) (left panel), pathways were identified based on CpGs in the final model. For telomere attrition rate (right panel), pathways were identified based on gene transcriptome correlated with the selected CpGs in the final model. Dot size indicates the number of genes (count) in the query list appearing in a pathway. Gene ratio of each pathway was calculated as the count divided by the total number of genes in the pathway. Dot color shows the over-representation analysis q-value.

40 of the 47 CpGs had gene name annotations, from which the pathway analysis suggested one significantly enriched biological pathway “translocation of SLC2A4 (GLUT4) to the plasma membrane” (Figure 4, left panel). Correlating the selected CpGs to RNA probes from the global gene expression microarray suggested 75 significantly correlated RNAs (p<0.05), which had no biological pathways significantly enriched.

### The DNAm-based explanatory model of telomere attrition rate in early childhood

### Selection of CpGs

Three CpGs were identified to associate with telomere attrition rate based on an FDR of 5% and are highlighted in Figure 2B. Only cg26557617 passed Bonferroni correction. 9,862 CpGs (p<0.01) were used for subsequent selection. 247 CpGs were then selected by an adaptive lasso model, based on which there were 30,381 pairwise interactions, summing up to 30,628 terms. The most relevant terms selected using an elastic net in at least 95% of the subsets were 25 CpGs and 5 interactions. Together with 6 CpGs which were involved in the selected interactions but not in the selected main effects, the final model contained 31 CpG main effects and 5 interactions. No overlap was observed between these terms and the model terms of baseline telomere length.

### The final model and interpretations

The Pearson correlation between telomere attrition rate and the weighted sum of the 36 terms was 0.87 and the R^2^ was 0.72. As is shown in Figure 3B and Supplementary Table 3, in the final model 18 of the 31 CpG main effects had telomere attrition rate-related increase in DNA methylation and 13 had inverse associations, also consistent with their EWAS associations. 7 CpGs were promoter-associated and 1 was related to a reprogramming-specific DMR (RDMR). 4 out of the 5 interactions had negative coefficients and the other had a positive coefficient.

25 CpGs in the final model were annotated with gene names but no pathway was found significantly enriched. Pathway analysis of the 54 RNA transcripts correlated to the selected 31 CpGs resulted in 30 enriched pathways (Figure 4, right panel) which are mostly related to signaling pathways in immune response.

## DISCUSSION

To our knowledge, the present study is the first to investigate the linkage of genome-wide DNA methylation profiles to newborn telomere length and early-life telomere attrition rate, incorporating interactions between epigenomic loci. Starting from whole-genome DNA methylation, a univariable CpG selection using EWAS in combination with a multivariable model construction using statistical learning was adopted to select relevant CpGs and CpG-CpG interactions. The selected DNAm-based explanatory models were distinct between baseline telomere length and telomere attrition rate, and a smaller number of CpGs were selected for telomere attrition rate than for baseline telomere length. Pathway analysis based on the identified CpGs suggested a role for GLUT4 translocation pathway in relation to baseline telomere length. Immune response-related cellular pathways were revealed in relation to telomere attrition rate in early childhood.

Two CpGs were identified strongly associated with baseline telomere length, one of which mapped to the promoter region of gene *PPM1F*, a gene encoding a protein phosphatase that negatively regulates stress response pathways [33]. Identifying this gene might explain the dependence of telomere length on cellular oxidative stress. However, this CpG was not selected in the final model, suggesting that the top CpGs in EWAS were not necessarily of the same importance in a multiple model. Indeed, only 8 of the 224 FDR significant CpGs from EWAS were in the final model of baseline telomere. CpGs in the final model suggested a pathway of translocating GLUT4 to plasma membrane through pathway enrichment analysis. In the meanwhile, two genes involved in this pathway, *TUBA1B* and *SLC2A4*, had their interacting CpGs selected in the final model. GLUT4 is a glucose transporter with a central role in glucose homeostasis and its translocation to plasma membrane is stimulated by insulin [34]. This might be in line with nutrient sensing as one of the hallmarks of aging [6] and supported by study findings that shorter telomere length was associated with type 2 diabetes mellitus [35] and insulin resistance [36]. Moreover, the pathway of GLUT4 translocation has been shown under the regulation of the same poly-ADP-ribosyl-transferase involved in telomere homeostasis [37]. Another CpG, cg13074173 (*RHPN1*), interacting with cg00973724 (*TUBA1B*), might also participate in this pathway since it regulates cytoskeleton activity [38] which is crucial for cellular translocation by vesicular transport.

The 35 CpGs selected in the final model of telomere attrition rate did not show significantly enriched pathways. Nevertheless, the top pathways (with q-value < 0.10) are collagen biosynthesis/formation, extracellular matrix (ECM) formation and N-lined glycosylation, all being related to ECM which plays an important role in tissue homeostasis [39] and is age-dependent [40]. 30 significantly enriched pathways were identified only after mapping to the cord blood transcriptome, with the majority pointing to T cell receptor signaling, NF-κB signaling and mitogen-activated protein (MAP) kinase signaling. Changes in immune system along with aging is termed as immunosenescence [41]. Active T cell signaling is accompanied by a high cell division rate and thus requires the support from telomere maintenance [42]. Transcription factor NF-κB, as a major driver of inflammatory signaling, is involved in several processes regulating telomere homeostasis, such as the modulation of telomerase activity and telomerase reverse transcriptase (TERT) transcription [43]. Additionally, telomere stability is attributed to the regulation by telomeric repeat binding factor 2 and TERT which are both under the control of MAP kinase pathways [44, 45].

Although a number of interaction terms were selected in both models, the number of interactions in the final models was much smaller than the number of main effects. It appears that the main effects play a more important role than their interactions in spite of the dependence between CpGs, which is in line with the idea of most published DNA methylation-based prediction models which only include CpG main effects.

The selected CpGs for the two outcomes showed an overlap only at the EWAS step (687 CpGs), and the final models did not have one predictor in common, possibly partly owing to the sample size and the conservative selection strategy applied. The discrepancy between baseline telomere length and telomere attrition rate in terms of CpG selection mirrors the discrepancy in epigenetic determinants of baseline telomere length and telomere dynamics that were set up at birth. Less CpGs and interactions were found to explain telomere attrition rate than baseline telomere length. This suggests that the initial setting of telomere length is more under epigenetic control compared to the change in telomere length thereafter. Indeed, there might be more external factors than just epigenetic and genetic factors at birth that can contribute to the change in telomere length after the *in utero* life, which may include lifestyle [46], psychological stress [47] and exposure to environmental pollutants [48–50]. Moreover, in the regard that the epigenome is also dynamic throughout life, identifying epigenetic predictors that contribute to telomere attrition might be warranted if DNA methylation is also profiled longitudinally.

Previously a 140-CpG DNAmTL estimator was published for adult telomere length [25] but did not overlap with the 47 CpGs selected for baseline telomere length in the present study. In addition, the 140-CpG estimator only weakly predicted cord blood telomere length in our study (r=0.088, p-value=0.10). Therefore, certain patterns of DNA methylation that are important at birth and early life may not be as important in later life for the regulation of telomere length, with both telomere length and DNA methylation being dynamic processes over life [2, 29].

A strength of this study is that we based our model on the genome-wide DNA methylation data from EPIC assay which is reliable and reproducible covering a wide range of genome regions [51], and supports a comprehensive search for relevant epigenetic biomarkers. In parallel, we assessed the associations for two outcomes, telomere length at birth and telomere attrition rate, providing a comparison between initial telomere length setting and telomere dynamics early in life. Secondly, in spite of a sample size that may not be sufficiently large and that lasso, as one of the components of elastic net, is inconsistent for variable selection [31], we used a step-wise selection in combination with a repeated sampling to overcome low statistical power and undesired selection bias. In addition, the statistical inference was performed by bootstrapping instead of parametric models to avoid violations to model assumption.

On the other hand, limitations of our study need to be acknowledged. First, real-time PCR was used to determine telomere length in newborns, which shows a higher inter-assay variation compared to the traditionally used terminal restriction fragments (TRF) method or flow-FISH method [52, 53], although an inter-laboratory comparison achieved a coefficient of variation less than 7%. The imperfect correlation between baseline cord blood telomere length and leukocyte telomere length could also be attributed to variation in measurement. This issue often raises an RTM effect in a repeated measurement situation, which was accounted for by applying an RTM-correction [54]. Among different proposed methods exploring telomere attrition in longitudinal settings, the RTM-correction method we applied has been shown not to yield biased effect estimates [55]. Secondly, a pre-screening by EWAS resulted in that not all CpGs in the DNA methylation array could participate in subsequent variable selection. Due to the correction of the test-statistics based on empirical null distribution and correction for multiple testing, EWAS is highly data-driven and unable to detect subtle associations for a quantitative trait such as telomere length. However, EWAS was useful in selecting CpGs that could be candidates for subsequent multivariable model building, being less computationally intense than direct multivariable selection in analyzing high dimensional data. Thirdly, although the influence of a small sample size was coped with as discussed above, our variable selection tended to be conservative since the shrinkage in elastic net often leads to a small magnitude in model parameter estimates. Fourthly, DNA methylation at certain loci could involve genomic imprinting which conveys parental regulation of gene expression, which is an interesting topic for future analyses since allele-specific information of the epigenetic markers are not available in the Infinium MethylationEPIC assay. Finally, we acknowledge that the current findings are explorative, and that external cohorts are needed to further validate our findings and to enable constructing a DNA methylation-based prediction model for newborn telomere length and early-life telomere attrition.

In conclusion, as a first analysis on both telomere length at birth and telomere attrition rate during early life, searching for polygenic explanatory DNA methylation, the present study has added to the evidence that the two aging biomarkers are associated, and identified potential relevant biological pathways. Especially, baseline telomere length and telomere attrition in early-life were identified with epigenetic signatures, and these epigenetic settings at birth contributed more to baseline telomere length than to telomere attrition.

## MATERIALS AND METHODS

### Study population

The ongoing prospective ENVIR*ON*AGE birth cohort [30] was initiated in 2010. This cohort study has been approved by the ethical committee of Hasselt University and East-Limburg Hospital (Genk, Belgium) (EudraCT B37120107805), and is carried out according to the Declaration of Helsinki.

The mother-newborn pairs were recruited at delivery in the East-Limburg Hospital and were invited for a follow-up visit when the children were around 4 years old. Cord blood samples were collected at birth and blood samples of the children were collected at the follow-up visit. Genome-wide DNA methylation profiled in cord blood was available in 372 samples. Among the 423 children attending the follow-up examination between October 2014 and October 2019, 247 children had matching cord blood (baseline) telomere length, leukocyte telomere length (follow-up) and cord blood DNA methylation. These samples were used to evaluate the relationship between DNA methylation and baseline telomere length or longitudinal change in telomere length from birth to childhood. The selected 247 participants are representative for the total ENVIR*ON*AGE population in terms of most characteristics as described in Table 1, but slightly differed in maternal education and newborn ethnicity (Supplementary Table 4).

Written informed consent was obtained from the participating mothers and questionnaires were filled out by the mothers to collect information on lifestyles and socioeconomic status. The information on newborns’ sex, birth weight and gestational age, and maternal age and parity was collected from medical records in the hospital. The date of conception was estimated based on the first day of the mother’s last menstrual period in combination with the first ultrasonographic examination. Maternal body mass index (BMI) was determined as the ratio of the maternal weight to the squared maternal height measured at the first antennal visit (weeks 7-9 of pregnancy). Ethnicity of a newborn was categorized as European if at least 2 grandparents were Europeans and classified as non-European otherwise. Educational level of the mothers was coded as 0 if they did not obtain any diploma, as 1 if they obtained at highest a high school diploma, as 2 if the highest diploma obtained was from a 3-year college and as 3 if obtained a 4-year college or university degree. Maternal smoking status was classified into “never smoker”, “former smoker” (when having quit smoking before pregnancy), or “smoker” (in case of continuing smoking during pregnancy). Mothers were regarded as with pregnancy complications if they had any of gestational diabetes, hypertension, hyper- or hypothyroidism, infectious disease, preeclampsia, vaginal bleeding, phenylketonuria and allergy or asthma during pregnancy.

### Blood sample collection and DNA extraction

Cord blood was collected immediately after delivery in BD Vacutainer® plastic whole blood tubes with spray-coated K2EDTA (BD, Franklin Lakes, NJ, USA). Samples were centrifuged at 3,200rpm for 15 minutes. Plasma was separated and the remainder with the buffy coats was stored at -80° C until further analysis. At the follow-up visit, blood was collected from the participating children by venipuncture using a winged steel needle in spray-coated K2EDTA tubes. After the sample process as described for cord blood, samples were stored at -80° C until analysis.

Cord blood and child blood DNA was extracted using the QIAamp DNA mini kit (Qiagen, Inc., Venlo, The Netherlands). DNA purity and concentration were verified by Nanodrop 1000 spectrophotometer (Isogen, Life Science, Belgium). DNA was considered pure when the A260/280 was greater than 1.80 and A260/230 greater than 2.0. DNA integrity was assessed with agarose gel electrophoresis.

### Average relative telomere length measurement and data processing

Average relative telomere length was measured in triplicates using a previously described quantitative, real-time polymerase chain reaction (qPCR) protocol [14]. Detailed assay specifications are provided in the Supplementary Method. Telomere length were expressed as the ratio of telomere copy number to single-copy gene number (T/S) relative to the average T/S ratio of the entire sample set. Assay-reliability was assessed using an intra-class correlation coefficient (ICC) [56]. The inter-assay ICC was 0.936 (95%CI: 0.808 to 0.969) and the intra-assay ICC was 0.952 (95%CI: 0.947 to 0.956).

The cord blood telomere lengths were measured along with the leukocyte telomere lengths at follow-up in order to ensure that matching samples of individuals were assayed under the same condition: samples from the same individuals were on the same qPCR plate. Having been measured in a single batch, the telomere length data did not undergo batch effect removal. Telomere attrition from birth to follow-up was defined as the difference between telomere length at birth and at follow-up, the latter subtracted from the former. The difference between telomere lengths at birth and at follow-up was assessed using a paired t-test. Whether the children had differential telomere length change (homogeneity) was tested using the test-statistic suggested by Berry et al [54]. To account for the RTM effect which might be a result of the variation in measurement, we adopted a previously published method [54] to obtain the RTM-corrected telomere attrition:

$$\Delta TL=\rho \theta (X_{1}-\mu_{1})-(X_{2}-\mu_{2})$$ (1)

where *X*_1_ and *X*_2_ are the notations for telomere length at birth and at follow-up, respectively, $\hat{\rho }=r$is the Pearson correlation between the telomere lengths measured at the two time points, and $\hat{\theta }=\frac{S_{2}}{S_{1}}$is the ratio of the standard deviation of telomere lengths at follow-up to the standard deviation of baseline telomere lengths. ${\hat{\mu }}_{1}=\bar{X_{1}}$ and ${\hat{\mu }}_{2}=\bar{X_{2}}$ denote the mean baseline telomere length and mean leukocyte telomere length at follow-up, respectively. Telomere attrition rate was calculated as telomere attrition divided by age (years) at follow-up.

### DNA methylation measurement and data processing

Cord blood DNA samples were bisulphite-converted, amplified and hybridized to the Illumina HumanMethylationEPIC Bead-Chip array (Illumina, San Diego, CA, USA), at the service lab GenomeScan (Leiden, The Netherlands). The array measurements were scanned using an Illumina iScan and the data quality was assessed using the R script MethylAid. DNA methylation data were preprocessed using the minfi package in R [57]. Briefly, probes with a call rate lower than 95% based on detection p-value > 10e-16 [58], samples with a call rate lower than 98% and samples with discordant sex, predicted using shinyMethyl [59], were removed. Methylation data was normalized using functional normalization [60]. For each CpG locus the methylation level was expressed as beta value calculated by M/(M+U) where M stands for the signal intensity from methylated probes and U stands for the signal intensity of unmethylated probes. The preprocessing resulted in a DNA methylation data with 857,898 CpGs. The missingness in the matrix of beta values was imputed by K-nearest neighbor (KNN) imputation and technical confounding effects (batch and position) were removed from the beta matrix using an empirical Bayes method [61]. Subsequently, we trimmed the data CpG-wisely for outliers which were lower than 3 inter-quarter-range below the 1^st^ quartile, or higher than 3 inter-quarter-range above the 3^rd^ quartile. CpG probes were filtered to exclude CpGs on X and Y chromosomes, those known to be common SNPs and those having cross-reactivity with multiple genomic locations [51]. 787,264 CpGs remained in the DNA methylation data were used in the EWAS of telomere lengths.

Based on the DNA methylation data, blood cell proportions (nucleated red blood cells, granulocytes, monocytes, natural killer cells, B cells, CD4^+^ T cells, and CD8^+^ T cells) in the cord blood samples were estimated using Bakulski algorithm [62].

### Pre-screening by EWAS

The design of the present study is illustrated in Figure 1. Since variable selection from the whole EPIC array is computationally expensive, we performed EWAS as a pre-screening for candidate CpGs. For the *j*-th CpG (*j*=1, 2, …, 787264), a multiple linear regression model was fitted to test the association between baseline telomere length (*Y_i_*, *i*=1, 2, …, 247) and the DNA methylation level at the CpG (*X_ij_*), with robust covariance matrix estimated using the R package *sandwich* [63, 64]:

$$Y_{i}=\beta_{0}+\beta_{m}X_{ij}+C_{i}^{T}\beta_{c}$$ (2)

CpGs entered models in their original scale without extra centering or standardization. Models were adjusted for covariates selected a priori (***C_i_***): newborn gestational age, sex, ethnicity and birth weight, maternal pre-pregnancy BMI, age, parity, medical condition during pregnancy and education, paternal age, and cell type heterogeneity. Similarly, an EWAS was performed for telomere attrition rate by fitting the same linear model as equation (2) for each CpG adjusting for the same set of covariates. The test-statistics of *β_m_* were corrected for an inflation factor and bias estimated based on their empirical null distribution [65], hence giving the corrected p-values. The Benjamini-Hochberg adjusted p-values were calculated based on the corrected p-values to identify significant EWAS probes (FDR=5%). In order to select a sufficiently large number of CpGs for the subsequent selection procedure, CpGs whose inflation- and bias-corrected p-value was smaller than a pre-determined threshold of 0.01 were the candidates that become the inputs of the next selection procedure.

### Variable selection by statistical learning

The variable selection in this study was within the framework of elastic net where CpG selection and estimation of the contributing weight of each predictor take place simultaneously. We aimed to build explanatory models that express the baseline telomere length or telomere attrition rate as a weighted sum of the methylation level of a set of CpGs. The variable selection method used for both outcomes were identical, therefore we only describe the method in detail for baseline telomere length here.

The aim of this study was to select a number of CpGs (*X_k_*) and CpG-CpG interactions (*X_s_X_t_*) that can express the *i*-th newborn’s baseline (cord blood) telomere length (*Y_i_*) as:

$$Y_{i}=w_{0}+\underset{k}{\sum }w_{k}X_{ik}+\underset{s,t}{\sum }v_{s,t}X_{is}X_{it}$$ (3)

where *w*_0_ is the model intercept, being the mean of *Y_i_* due to the standardization of CpGs. Parameters *w_k_* and *v_s,t_* denote the contributing weights of the CpGs and interaction terms in the model. The model was trained with the matrix of candidate CpGs as the inputs and the vector of baseline telomere lengths as the output. In order to reduce the influence by the difference between CpG’s magnitude and possible collinearity, the CpGs were standardized to have a mean of 0 and standard deviation of 1 before entering the model. When interactions are subject to selection, the pairwise interaction of CpGs were made by multiplying pairwisely the standardized CpGs.

The elastic net regression minimizes the objective function (simplified to include only main effects)

$$\begin{array}{l} \frac{1}{2N}\sum_{i=1}^{N}{\left(Y_{i}-\underset{k}{\sum^{\text{​}}}w_{k}X_{ik}\right)}^{2}\\ \;\;\;\;\;\;\;\;\;\;\;\;\;\;\;\;\;\;\;\;\;+\left[\lambda \frac{1}{2}(1-\alpha )\underset{k}{\sum^{\text{​}}}w_{k}{}^{2}+\alpha \underset{k}{\sum^{\text{​}}}|w_{k}|\right] \end{array}$$ (4)

where *N* is the number of observations. The second term is the penalty on coefficient estimates of variables (*w_k_*). The regularization parameter *λ* was determined by 10-fold cross validation based on model deviance. In order to balance between model predictability and model overfitting, the *λ* value corresponding to a 1*σ* deviation (*λ_1σ_*) from *λ=λ_min_* was chosen, which led to a slightly smaller number of predictors than with the *λ_min_*. The tuning parameter *α* between lasso (*α*=1) and ridge (*α*=0) regression was determined by computation over a sequence of α value ranging from 0 to 1 with step of 0.05 and the *α* with the smallest deviance was selected. The models were fit in R with package *glmnet* [66], where coordinate descent algorithm was used and convergence threshold was set to 10^-7^.

Adaptive lasso, a consistent penalized model, variable selection, was used to select candidate CpGs in a multiple linear model including all 22,817 CpG’s selected from pre-screening by EWAS. The adaptive lasso performs lasso regression (*α*=1) by shrinking coefficients of less relevant variables to zero, while at the same time assigning unequal penalties to each variable [31]. The penalty term shown in equation (4) for adaptive lasso was *λ*∑*_k_ a_k_* |*w_k_*|, where *a_k_* was the penalty weight for the *k*-th CpG and was determined in a data-driven way as the inverse of the absolute value of the ridge regression coefficient of the corresponding CpG, as the ridge regression accounts for correlations among variables. By doing so, the CpGs given more shrinkage in ridge regression were assigned even higher penalty in the adaptive lasso variable selection. 208 CpGs were selected by adaptive lasso, which forms 21,528 pairwise interactions. Thus, a total number of 21,736 terms underwent the elastic net selection.

To encounter the issue that one of the components of elastic net, the lasso penalty, is not statistically consistent, we used 1000 repeatedly randomly sampled subsets from the whole data set for model selection. During each round, 85% of the samples (n=210) were taken and used to fit an elastic net, for which the parameters *α*, *λ_1σ_* and the number of predictors corresponding to the selected model were recorded. Among all the CpG and interactions, those has been selected in no less than 95% of the 1000 subsets were regarded as active terms and put in the final model. The weights *w_k_* in equation (3) were estimated by fitting a ridge regression model on all the selected CpGs and interactions to the whole data set. Since statistical inference was not available within the ridge regression framework, the p-values of the ridge coefficients were simulated via bootstrap with 10,000 replicates.

### Functional annotation of the selected CpGs

The selected CpGs were annotated with genomic regions (promoter region or DMRs) and UCSC reference gene names. Chord diagram visualization was performed using R package *circlize* [67].

To assist the translation of the identified CpGs into biological pathways, the DNA methylation level of the CpGs were correlated to the gene expression data available in the ENVIR*ON*AGE birth cohort. Global gene expression profiling by microarrays was performed in cord blood samples using a method described previously [68]. 44 samples which were available in both DNA methylation and global gene expression profiles were used in searching for RNA probes that are correlated with the selected CpGs. The correlation was assessed by computing the Pearson correlation coefficient within each CpG-RNA pair, which were defined based on gene name annotation or genomic location. Among the 47 CpGs explaining baseline telomere length, 36 CpGs found RNA probes with the same gene name annotated. Additionally, each of the 47 CpGs was correlated to the RNA transcripts on the same chromosome whose starting sites were within 1Mb from the CpG locus. In total, 1,311 correlations were calculated and 75 correlations had p-value<0.05. The gene names corresponding to the 75 correlated RNA transcripts were used for a pathway analysis. Similarly, there were 676 RNA transcripts correlated to the 31 CpGs explaining telomere attrition rate: 22 were found based on gene name and 654 were found within genomic neighborhood. 53 correlations with p-value < 0.05 were used for a pathway analysis.

The gene names of selected CpGs and the correlated transcripts, respectively, were mapped to the Reactome pathway database [69] using the R package *ReactomePA* [70] for an over-representation analysis (ORA). A pathway was considered significantly enriched if its q-value was smaller than FDR of 5% and at least 3 genes were involved in the pathway.

### Ethics approval and consent to participate

The study protocol was approved by the Ethical Committee of Hasselt University and East-Limburg Hospital in Genk (Belgium) and has been carried out according to the declaration of Helsinki. Written informed consent was obtained from all participating mothers.

### Data availability

Data are available on request.

## Supplementary Material

Supplementary Method

Supplementary Figure 1

Supplementary Tables
