# Supplementary material for: Different epigenetic signatures of newborn telomere length and telomere attrition rate in early life
Source: Aging (Albany NY). 2021 Jun 4;13(11):14630–50. doi: 10.18632/aging.203117 (PMC8221291; doi:10.18632/aging.203117)
Supplement: Supplementary Method [file aging-13-203117-s001.pdf]

SUPPLEMENTARY METHOD

Telomere length measurement by qPCR assay

DNA samples were normalized to ensure a uniform DNA input of 5 ng for each qPCR, and this was checked using the Quant-iT™ PicoGreen® dsDNA Assay Kit (Life Technologies, Europe). Cord blood and child blood samples were matched per individual and arranged on the same qPCR plate and were measured in 1 batch. All samples were measured in triplicates on a 7900HT Fast RealTime PCR System (Applied Biosystems) in a 384-well format. The telomere-specific qPCR reaction mixture contained 1x QuantiTect SYBR Green PCR master mix (Qiagen, Inc., Venlo, the Netherlands), 2 mM dithiothreitol (DTT), 300 nM telg primer (ACACTAAGGTTTGGGT TTGGGTTTGGGTTTGGGTTAGTG T) and 900 nM telc primer (TGTTAGGTATCCCTATCCCTATCCCT ATCCCTATCCCTAACA). Used cycling conditions were: 1 cycle at 95° C for 10 min, 2 cycles at 94° C for 15 sec and 49° C for 2 min, and 30 cycles at 94° C for 15 sec, 62° C for 20 sec, and 74° C for 1 min and 40 sec. The single-copy gene (human β globin) qPCR mixture contained 1x QuantiTect SYBR Green PCR master mix, 400 nM HBG1 primer (GCTTCTGACACA ACTGTGTTCACTAGC) and 400 nM HBG2 primer (CACCAACTTCATCCACGTTCACC). Used cycling conditions were: 1 cycle at 95° C for 10 min, 40 cycles at 95° C for 15 sec, and 58° C for 1 min and 20 sec. After each qPCR a melting curve analysis was performed. In each run, a 6-point serial dilution of pooled DNA was run to assess PCR efficiency. PCR-efficiencies ranged from 100-105% for telomere runs and 95-100% for single-copy gene runs. Furthermore 10 inter-run calibrators (IRCs) were run to account for inter-run variability over 16 qPCR plates. qPCR curves for each sample were visually inspected and when technical problems were detected or triplicates showed too high variability, samples were excluded for further analysis. Telomeres were normalized using qBase (Biogazelle, Zwijnaarde, Belgium). The reliability of our assay was assessed by calculating the intraclass coefficient (ICC) with 95% CI of triplicate measures (T/S ratios) [1]. Both the inter-assay (based on 10 IRCs over 16 qPCR plates) and intra-assay ICC (based on all measures) was calculated using the available on-line R script on the Telomere Research Network website [2].

REFERENCES

1. Telomere research network: Study Design and Analysis. 2020. Available from: https://trn.tulane.edu/resources/study-design-analysis/

2. Eisenberg D, Nettle D, Verhulst S. How to calculate the repeatability (ICC) of telomere length measures. 2020. Available from: https://trn.tulane.edu/wp-content/uploads/sites/445/2020/10/How-to-calculate-repeatability.pdf
