# Supplementary material for: Different epigenetic signatures of newborn telomere length and telomere attrition rate in early life
Source: Aging (Albany NY). 2021 Jun 4;13(11):14630–50. doi: 10.18632/aging.203117 (PMC8221291; doi:10.18632/aging.203117)
Supplement: Supplementary Figure 1 [file aging-13-203117-s002.pdf]

SUPPLEMENTARY FIGURE

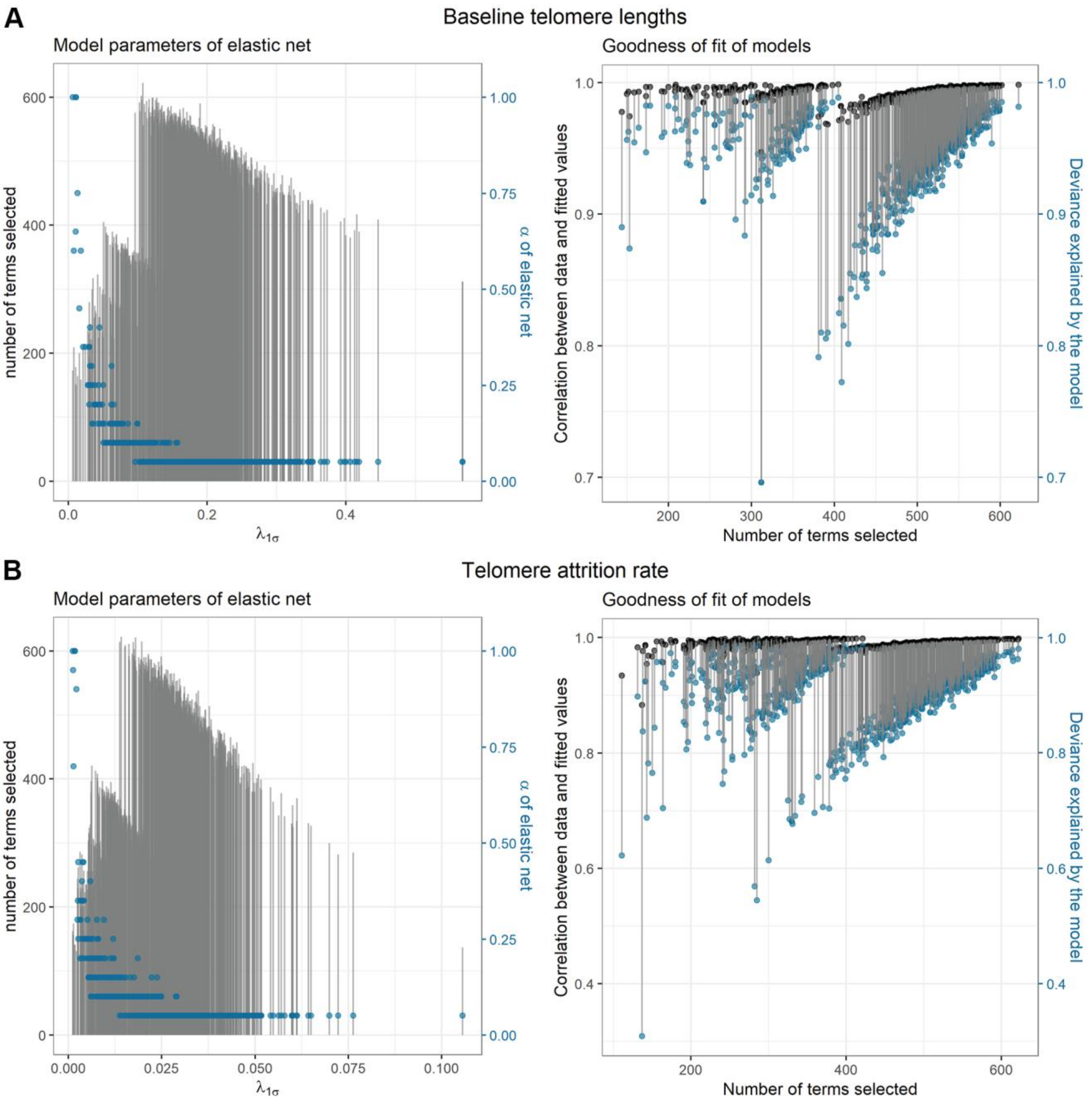

Supplementary Figure 1. Parameters of elastic net model selection and model goodness-of-fit. Sub-figures in left show that the 1000 randomly sampled subsets are distinct subsets with various regularization parameters: the length of grey segments represents the number of terms selected, and the blue dots show the optimal α values picked for each subset (ranging from 0.05 to 1). Sub-figures in the right column show the fitting performance of each selected model in the corresponding subsets with the black dots depicting the Pearson correlation between telomere length or telomere attrition and the estimation made based on each selected model. The deviance explained by model (equivalent to model R^2) is shown with blue dots. For baseline telomere lengths (**A**), the median of the parameter α was 0.05. $\lambda_{1\sigma}$ ranged from 0.0067 to 0.57 with a median of 0.18. The smallest number of selected terms was 144 (including 51 main effects and 93 interactions) while the largest was 622 (including 154 main effects and 468 interactions). Models including larger numbers of predictors showed better fitting performance. For telomere attrition rate (**B**), the parameter α had a median of 0.05 and $\lambda_{1\sigma}$ ranged from 0.0012 to 0.11 with a median of 0.025. The smallest number of selected terms was 111 (including 47 main effects and 64 interactions) while the largest was 622 (including 173 main effects and 449 interactions).
