# Supplementary material for: Different epigenetic signatures of newborn telomere length and telomere attrition rate in early life
Source: Aging (Albany NY). 2021 Jun 4;13(11):14630–50. doi: 10.18632/aging.203117 (PMC8221291; doi:10.18632/aging.203117)
Supplement: Supplementary Tables [file aging-13-203117-s003.pdf]

SUPPLEMENTARY TABLES

Supplementary Table 1. The summary of the pairwise Pearson correlations of the CpGs which passed pre-screening by an epigenome-wide association analysis.

	min	5th percentile	Median	95th percentile	max
**Cord blood telomere length [1]**	-0.95	-0.19	0.05	0.78	0.99
**Telomere attrition rate [2]**	-0.96	-0.28	0.03	0.49	0.98

[1]260,296,336 pairwise Pearson correlations of 22,817 CpGs.
[2]48,624,591 pairwise Pearson correlations of 9,862 CpGs.

Supplementary Table 2. The 47 CpGs and 7 interactions in the model of baseline telomere length.

Name of CpG or interaction	Coefficient (bootstrap p-value)	EWAS Coefficient (p-value)	Chrom-osome	Position on chromosome	Relation to island	Gene name	DMR	Promoter associated
**(intercept)**	1.1605 (0.00e+00)							
**cg00036011**	0.0169 (4.00e-04)	20.68 (9.17e-04)	chr6	26271718	N_Shore	HIST1H3G; HIST1H2BI		
**cg00064950**	-0.0135 (1.10e-03)	-8.51 (4.32e-03)	chr17	7184216	Island	SLC2A4		
**cg00487989**	0.0177 (7.00e-04)	7.64 (7.07e-04)	chr16	89688244	OpenSea	DPEP1		
**cg00871560**	0.0112 (1.12e-02)	1.47 (2.32e-04)	chr3	132756721	N_Shore	TMEM108		
**cg00973724**	0.0109 (1.18e-02)	7.43 (2.76e-03)	chr12	49524797	Island	TUBA1B		yes
**cg00973724:cg00064950**	-0.0086 (2.66e-02)					*TUBA1B * SLC2A4*		
**cg01567315**	0.0156 (1.10e-03)	4.52 (2.60e-04)	chr1	155650264	OpenSea	YY1AP1		
**cg02576753**	0.0132 (4.40e-03)	1.56 (2.56e-06)	chr15	27772640	OpenSea	GABRG3		
**cg02802645**	-0.0139 (5.60e-03)	-6.17 (3.64e-03)	chr22	38904757	S_Shore			
**cg03891344**	-0.0193 (4.00e-04)	-9.98 (1.25e-04)	chr16	19533212	Island	GDE1		yes
**cg04372430**	-0.0074 (7.54e-02)	-9.06 (6.31e-03)	chr8	48591406	OpenSea	SPIDR		
**cg04372430:cg16435922**	-0.0171 (2.00e-04)							
**cg06105699**	0.0162 (4.00e-04)	1.15 (2.53e-03)	chr17	79971436	OpenSea	ASPSCR1		
**cg06529600**	-0.0066 (1.18e-01)	-2.15 (2.60e-03)	chr6	112652308	OpenSea			
**cg06826039**	-0.0099 (2.82e-02)	-6.65 (3.07e-04)	chr1	204379650	N_Shore	PPP1R15B		yes
**cg07131116**	0.0113 (2.57e-02)	9.4 (2.34e-06)	chr14	94546722	N_Shore	DDX24; IFI27L1		yes
**cg07417551**	0.0108 (1.75e-02)	3.34 (3.30e-03)	chr19	14586295	S_Shore	PTGER1		
**cg08878914**	-0.0099 (2.53e-02)	-5.9 (4.85e-03)	chr8	86435445	OpenSea			
**cg09793256**	-0.0112 (9.80e-03)	-7.08 (4.66e-04)	chr2	55646604	N_Shore	CCDC88A	DMR	yes
**cg09880724**	-0.0142 (1.30e-03)	-34.01 (3.75e-03)	chr16	87425644	Island	FBXO31; MAP1LC3B		yes
**cg11300971**	0.0143 (4.10e-03)	1.88 (1.33e-04)	chr4	89619038	OpenSea	NAP1L5; HERC3		yes
**cg11455582**	-0.0129 (3.90e-03)	-8.57 (7.86e-03)	chr3	155272990	OpenSea	PLCH1		
**cg12581244**	0.0091 (6.35e-02)	4.65 (2.06e-03)	chr17	33700295	N_Shore	SLFN11		yes
**cg13074173**	-0.0074 (7.85e-02)	-3.27 (4.55e-03)	chr8	144462007	Island	RHPN1		
**cg13074173:cg00973724**	-0.0146 (2.20e-03)					*RHPN1 * TUBA1B*		
**cg14055864**	-0.0129 (7.50e-03)	-7.72 (3.72e-03)	chr3	138067120	Island	MRAS		
**cg14102251**	0.0062 (1.46e-01)	6.08 (7.67e-03)	chr14	75745098	Island	FOS		
**cg14102251:cg12581244**	0.0101 (2.70e-02)					*FOS * SLFN11*		
**cg14264119**	0.0169 (0.00e+00)	2.37 (5.41e-05)	chr10	11783794	N_Shore	ECHDC3		
**cg14493336**	0.0085 (3.73e-02)	0.68 (5.11e-03)	chr2	65295115	OpenSea	CEP68		
**cg14493336:cg14264119**	0.0091 (1.50e-02)					*CEP68 * ECHDC3*		
**cg15380694**	0.0125 (1.06e-02)	9.52 (3.49e-04)	chr8	125577065	OpenSea	MTSS1		yes
**cg15661580**	-0.0132 (6.00e-03)	-3.82 (1.04e-03)	chr10	71896835	OpenSea			
**cg15821589**	-0.0169 (2.00e-04)	-2.89 (8.26e-06)	chr1	78444904	Island	FUBP1		
**cg16435922**	0.016 (6.00e-04)	3.84 (4.55e-05)	chr16	55053053	Island			
**cg17282982**	0.0161 (0.00e+00)	34.56 (4.40e-05)	chr1	114355064	Island	RSBN1		yes
**cg17332573**	0.0126 (3.10e-03)	10.68 (1.22e-06)	chr6	26250934	N_Shore	HIST1H2BH; IST1H3F		yes
**cg19822755**	-0.0108 (7.60e-03)	-1.95 (1.97e-04)	chr16	30108259	S_Shore	YPEL3		
**cg20249169**	0.0128 (6.60e-03)	10.23 (2.72e-03)	chr2	64881356	Island	SERTAD2		

**cg20249169:cg07417551**	0.0191 (1.00e-03)					*SERTAD2 * PTGER1*	
**cg20467667**	0.0127 (6.10e-03)	15.65 (1.32e-06)	chr10	75987922	OpenSea	ADK	yes
**cg21954346**	0.0126 (5.80e-03)	8.03 (2.26e-04)	chr16	68271489	Island	ESRP2	yes
**cg22069262**	-0.0095 (4.73e-02)	-3.66 (6.60e-03)	chr7	1587402	Island	TMEM184A	
**cg22335713**	0.0106 (1.36e-02)	4.78 (3.94e-04)	chr10	123357603	Island	FGFR2	
**cg22335713:cg22577984**	-0.0157 (5.10e-03)					*FGFR2 * C2CD2L*	
**cg22577984**	-0.011 (1.83e-02)	-2.86 (5.99e-03)	chr11	118981363	S_Shelf	C2CD2L	
**cg22749642**	0.012 (4.70e-03)	1.29 (6.78e-03)	chr11	857033	N_Shore	TSPAN4	
**cg23455212**	-0.0056 (2.24e-01)	-1.7 (8.00e-03)	chr12	91505417	OpenSea	LUM	
**cg24803876**	0.0112 (1.07e-02)	12.81 (7.11e-04)	chr8	67340974	Island	RRS1	
**cg26035817**	-0.0115 (1.70e-02)	-1.89 (7.30e-04)	chr1	114414802	OpenSea	PTPN22	yes
**cg26110653**	-0.0139 (3.00e-04)	-6.7 (7.53e-06)	chr13	38924412	Island	UFM1	yes
**cg26725552**	-0.0143 (2.40e-03)	-2.77 (1.73e-03)	chr10	126314758	OpenSea	FAM53B	
**cg26795934**	0.0079 (1.01e-01)	23.3 (7.15e-06)	chr11	58421700	OpenSea		
**ch.7.1140112F**	0.0105 (3.39e-02)	10.2 (1.28e-05)	chr7	49032475	OpenSea		

From left to right column: names of the CpGs or CpG-CpG interactions, coefficients (contributing weights) of each CpG in the final model with bootstrap p-value, the fully adjusted association and corrected p-value obtained in epigenome-wide association analysis (EWAS), the chromosome where the CpGs were located and the genes to which the CpGs were mapped. Gene names of interaction terms (two gene names in italic and separated by "*") indicate that both interacting CpGs were annotated with gene names.

Supplementary Table 3. The 31 CpGs and 5 interactions in the model of telomere attrition rate.

Name of CpG or interaction	Coefficient (bootstrap p-value)	EWAS Coefficient (p-value)	Chromosome	Position on chromosome	Relation to island	Gene name	DMR	Promoter associated
**(intercept)**	8e-04 (4.62e-01)							
**cg02537587**	0.002 (1.90e-03)	1.24 (3.32e-05)	chr12	3982809	S_Shore	PARP11		yes
**cg02690648**	0.0024 (1.00e-04)	1.93 (1.49e-06)	chr8	10683044	OpenSea	PINX1; MIR1322		
**cg04043642**	-0.0024 (4.00e-04)	-0.17 (6.00e-03)	chr20	55842871	S_Shore	BMP7		
**cg04935434**	0.0021 (2.10e-03)	1.12 (3.83e-03)	chr2	177024947	Island			
**cg05201958**	0.002 (7.00e-04)	0.14 (1.34e-03)	chr10	61968731	OpenSea	ANK3		
**cg06107116**	0.0019 (1.00e-03)	1.31 (6.96e-05)	chr6	33267515	Island	TAPBP; RGL2		yes
**cg08134068**	-0.0022 (3.00e-04)	-1.37 (9.09e-03)	chr7	151039078	Island	NUB1		yes
**cg09366730**	-0.0021 (1.90e-03)	-0.2 (4.61e-06)	chr4	2341370	Island	ZFYVE28		
**cg10020325**	0.0016 (9.90e-03)	0.17 (7.77e-03)	chr2	220362163	Island	GMPPA		yes
**cg10020325:cg16798728**	-0.0025 (1.00e-04)					*GMPPA * DPP6*		
**cg11086312**	0.002 (1.60e-03)	0.1 (9.34e-04)	chr14	92720273	OpenSea			
**cg11872375**	0.0024 (3.00e-04)	0.7 (3.43e-04)	chr12	124709358	OpenSea	ZNF664-FAM101A		
**cg12267637**	0.0018 (2.90e-03)	0.15 (7.96e-04)	chr2	4102455	OpenSea			
**cg12267637:cg23423086**	-0.0017 (4.20e-03)							
**cg12782249**	-0.0017 (4.50e-03)	-0.07 (6.00e-04)	chr15	91458455	OpenSea	MAN2A2		
**cg13960352**	0.0017 (3.10e-03)	0.59 (4.79e-06)	chr19	45826863	Island	CKM		
**cg15072796**	0.0015 (1.40e-02)	1.31 (9.86e-04)	chr10	105727772	S_Shore	SLK		
**cg16467775**	-0.0017 (4.30e-03)	-0.2 (7.31e-04)	chr16	90085110	Island	DBNDD1		
**cg16718624**	-0.0016 (3.70e-03)	-3.55 (1.38e-03)	chr3	77089518	S_Shore	ROBO2		
**cg16798728**	-0.0015 (7.50e-03)	-0.22 (7.03e-03)	chr7	153984755	OpenSea	DPP6		
**cg16798728:cg15072796**	-0.0022 (3.00e-04)					*DPP6 * SLK*		
**cg16975599**	0.0017 (7.40e-03)	0.15 (4.88e-05)	chr3	158962761	OpenSea	IQCJ		
**cg17751153**	-0.0021 (1.00e-03)	-0.39 (3.51e-04)	chr5	447880	OpenSea	EXOC3		yes
**cg19027504**	0.0025 (0.00e+00)	1.23 (1.39e-04)	chr12	40013700	OpenSea	ABCD2		yes
**cg19291087**	0.002 (1.00e-03)	1.27 (1.67e-04)	chr2	3502760	N_Shore	ADI1		
**cg19291087:cg04935434**	-0.0022 (4.00e-04)							
**cg19291087:cg16718624**	0.0021 (5.00e-04)					*ADI1 * ROBO2*		
**cg19887293**	-0.0015 (8.80e-03)	-0.2 (1.19e-03)	chr6	33132693	S_Shelf	COL11A2		
**cg20054536**	-0.0027 (7.00e-04)	-0.27 (1.40e-05)	chr19	376069	Island	THEG		

**cg23155965**	0.0021 (5.00e-04)	0.11 (2.70e-03)	chr10	134778176	N_Shore			
**cg23423086**	0.0017 (6.50e-03)	0.58 (1.24e-03)	chr11	85856245	OpenSea			
**cg23965459**	-0.0025 (0.00e+00)	-0.52 (3.71e-04)	chr14	104647094	S_Shore	KIF26A		
**cg24101053**	0.0021 (1.00e-03)	0.56 (1.03e-03)	chr5	14419738	OpenSea	TRIO		
**cg24790788**	-0.0015 (8.60e-03)	-0.1 (1.05e-03)	chr3	145879626	S_Shore	PLOD2	RDMR	
**cg24942922**	-8e-04 (1.28e-01)	-0.17 (2.41e-04)	chr3	120003547	N_Shore			
**cg25615010**	0.0013 (3.78e-02)	1.9 (7.57e-03)	chr7	152456741	Island	ACTR3B		yes

From left to right column: names of the CpGs or CpG-CpG interactions, coefficients (contributing weights) of each CpG in the final model with bootstrap p-value, the fully adjusted association and corrected p-value obtained in epigenome-wide association analysis (EWAS), the chromosome where the CpGs were located and the genes to which the CpGs were mapped. DMR: differentially methylated region. RDMR: reprogramming-specific DMR. Gene names of interaction terms (two gene names in italic and separated by "*") indicate that both interacting CpGs were annotated with gene names.

Supplementary Table 4. Comparison of characteristics of the selected with the total data in ENVIR*ON*AGE cohort.

		Selected data (N=247)	Total data (N=916)
Newborns			
Sex	Female	121 (49.0%)	459 (50.1%)
Ethnicity	European	231 (93.5%)	808 (88.2%)
Birth weight (kg)		3.39 ± 0.44	3.39 ± 0.48
Gestational age (weeks)		39.24 ± 1.37	39.13 ± 1.58
Mothers			
Education			
	No diploma	24 (9.7%)	115 (12.6%)
	High school diploma	67 (27.1%)	333 (36.4%)
	A 3-year college	124 (50.2%)	354 (38.6%)
	A 4-year college or university	32 (13.0%)	114 (12.4%)
Smoking status			
	Never smoker	167(67.6%)	582 (63.5%)
	Former smoker	48 (19.4%)	210 (22.9%)
	Smoker	32 (13.0%)	124 (13.5%)
Parity			
	Primiparous	129 (52.2%)	503 (54.9%)
	Secundiparous	95 (38.5%)	313 (34.2%)
	Multiparous	23 (9.3%)	100 (10.9%)
With pregnancy complications		37 (15.0%)	166 (18.1%)
Age at delivery (years)		30.21 ± 4.38	29.29 ± 4.59
Pre-pregnancy BMI (kg/m2)		24.28 ± 4.56	24.26 ± 4.54
Fathers			
Age at the mother's delivery (years)		32.87 ± 5.60	31.88 ± 5.52
